# Health Care Professionals’ Experience of Using AI: Systematic Review With Narrative Synthesis

**DOI:** 10.2196/55766

**Published:** 2024-10-30

**Authors:** Abimbola Ayorinde, Daniel Opoku Mensah, Julia Walsh, Iman Ghosh, Siti Aishah Ibrahim, Jeffry Hogg, Niels Peek, Frances Griffiths

**Affiliations:** 1 Division of Health Sciences Warwick Medical School University of Warwick Coventry United Kingdom; 2 AI Digital Health Research and Policy Group University Hospitals Birmingham NHS Foundation Trust Birmingham United Kingdom; 3 Division of Informatics, Imaging and Data Sciences Faculty of Biology, Medicine and Health University of Manchester Manchester United Kingdom; 4 The Healthcare Improvement Studies Institute Department of Public Health and Primary Care University of Cambridge Cambridge United Kingdom

**Keywords:** artificial intelligence, clinical decision support systems, CDSS, decision-making, quality assessment, clinician experience, health care professionals, health care delivery

## Abstract

**Background:**

There has been a substantial increase in the development of artificial intelligence (AI) tools for clinical decision support. Historically, these were mostly knowledge-based systems, but recent advances include non–knowledge-based systems using some form of machine learning. The ability of health care professionals to trust technology and understand how it benefits patients or improves care delivery is known to be important for their adoption of that technology. For non–knowledge-based AI tools for clinical decision support, these issues are poorly understood.

**Objective:**

The aim of this study is to qualitatively synthesize evidence on the experiences of health care professionals in routinely using non–knowledge-based AI tools to support their clinical decision-making.

**Methods:**

In June 2023, we searched 4 electronic databases, MEDLINE, Embase, CINAHL, and Web of Science, with no language or date limit. We also contacted relevant experts and searched reference lists of the included studies. We included studies of any design that reported the experiences of health care professionals using non–knowledge-based systems for clinical decision support in their work settings. We completed double independent quality assessment for all included studies using the Mixed Methods Appraisal Tool. We used a theoretically informed thematic approach to synthesize the findings.

**Results:**

After screening 7552 titles and 182 full-text articles, we included 25 studies conducted in 9 different countries. Most of the included studies were qualitative (n=13), and the remaining were quantitative (n=9) and mixed methods (n=3). Overall, we identified 7 themes: health care professionals’ understanding of AI applications, level of trust and confidence in AI tools, judging the value added by AI, data availability and limitations of AI, time and competing priorities, concern about governance, and collaboration to facilitate the implementation and use of AI. The most frequently occurring are the first 3 themes. For example, many studies reported that health care professionals were concerned about not understanding the AI outputs or the rationale behind them. There were issues with confidence in the accuracy of the AI applications and their recommendations. Some health care professionals believed that AI provided added value and improved decision-making, and some reported that it only served as a confirmation of their clinical judgment, while others did not find it useful at all.

**Conclusions:**

Our review identified several important issues documented in various studies on health care professionals’ use of AI tools in real-world health care settings. Opinions of health care professionals regarding the added value of AI tools for supporting clinical decision-making varied widely, and many professionals had concerns about their understanding of and trust in this technology. The findings of this review emphasize the need for concerted efforts to optimize the integration of AI tools in real-world health care settings.

**Trial Registration:**

PROSPERO CRD42022336359; https://tinyurl.com/2yunvkmb

## Introduction

Artificial intelligence (AI) refers to the ability of machine or computer programs to replicate human intelligence. AI is widely believed to have considerable potential in health care, ranging from the transformation of many aspects of patient care to streamlining administrative processes within and between various organizations involved in health care [1]. Specific potential benefits include earlier detection of disease, improved patient safety, improved estimation of capacity needs, and the facilitation of personalized medicine [1,2]. Globally, there has been great interest in the use of AI in health care settings [3]. For example, the UK government invested £250 million (US $328 million) in a national laboratory to boost AI in the National Health Service in 2019 [2].

AI has various applications in health care, but one of the most well-known applications involves using AI to enhance health care delivery to aid clinical decision-making in a process known as clinical decision support systems (CDSSs) [4]. Historically, CDSSs matched individual patient characteristics to a manually curated, computerized clinical knowledge base [5]. These “knowledge-based” CDSSs comprised a set of “if and then” rules with the system retrieving data from the knowledge base to evaluate the rule and then generating an output or suggested action [4]. More recently, machine learning methods have enabled the development of “non–knowledge-based” systems that use complex representations (patterns, trees, networks, and equations) derived from large datasets to generate outputs or actions [4]. Machine learning models are typically difficult to understand for humans because of their complexity, especially when they have been derived via deep learning, which is the most common method nowadays [4].

The integration of this form of AI tools in health care is notably progressing more slowly than initially anticipated, given the levels of investment, due to several challenges to implementation [6,7]. Some of these challenges extend beyond a specific domain of AI tools and reflect broader issues encountered in the implementation of innovation in practice. Frameworks such as the Consolidated Framework for Implementation Research shed light on these challenges by emphasizing the multifaceted nature of the implementation process [8]. Many challenges in AI implementation are similar to those encountered in the implementation of any new digital technology, and several frameworks have provided comprehensive lens through which these challenges can be examined, for example, the Non-adoption, Abandonment, Scale-up, Spread and Sustainability framework [9] and the meta-analysis–based modified Unified Theory of Acceptance and Use of Technology (meta-UTAUT) [10,11]. However, issues specific to the use of AI technologies, particularly the non–knowledge-based systems have been reported [7]. Health care professionals often struggle with concerns about the reliability, interpretability, and ethical implications of these AI systems [6,7].

This review aimed to qualitatively synthesize the existing evidence on the experiences of health care professionals in using non–knowledge-based CDSSs (referred to as AI tools in the Methods and Results sections) that are deployed in their work setting with a focus on issues specific to non–knowledge-based CDSSs. We believe that focusing on issues specific for non–knowledge-based CDSSs will help with understanding the challenges that remain for the deployment of these systems in health care.

## Methods

### Search Strategy

This reporting of this systematic review adheres to the PRISMA (Preferred Reporting Items for Systematic Reviews and Meta-Analyses) reporting guideline because the PRISMA-AI (Preferred Reporting Items for Systematic Reviews and Meta-Analyses Artificial Intelligence) guideline was not available at the time of writing [12,13]. We conducted a systematic search of the MEDLINE, Embase, CINAHL, and Web of Science databases for relevant articles. Searches were initially conducted in May 2022, and the searches were updated in June 2023. Overall, 4 key concepts and their possible variations informed the search strategy: AI (such as artificial intelligence, machine learning, and natural language processing); decision support systems (such as decision support system and computer-assisted diagnosis); health care professionals (such as health care professionals, nurse, and physician); and terms relating to experience (such as experience, view, and opinion). Terms within similar categories were combined with OR and then the results from each category were combined with AND (Multimedia Appendix 1). The reference lists of eligible studies were also screened, and experts were contacted for relevant articles that may have been missed. There were no language and date restrictions.

### Study Selection

The search results were imported into EndNote (Clarivate Analytics). referencing and bibliography management software to remove duplicates. The titles and abstracts of the retrieved articles were screened by 1 reviewer (AA, JW, or DOM), and a random sample of 20% (1511/7552) of the retrieved articles were screened by a second reviewer. The title and abstract screening was completed on Rayyan software (Rayyan), a web-based application for systematic reviews. The full texts of potentially relevant articles were then retrieved and examined in detail for eligibility by 2 independent reviewers (AA and IG or JW and DOM). Any discrepancies were resolved by discussion between reviewers and a third reviewer, or the whole project team was consulted when necessary. The most challenging aspect of the study selection was deciding whether the AI described in that particular study is knowledge based or non–knowledge based. When this was unclear, we discussed as a team and deferred to the team member with data science expertise (NP) for final decision.

### Inclusion and Exclusion Criteria

Studies were included if (1) they targeted health care or social care professionals; (2) the article described a non–knowledge-based CDSSs deployed for health care; and (3) the study explored health care professionals’ experiences of using AI tools in health care or barriers and facilitators to AI tool use in the real world. All study designs were deemed acceptable for inclusion, but nonprimary studies were excluded. However, we screened the references included in the relevant reviews to identify primary studies that may have been missed from our searches. The inclusion and exclusion criteria for study selection are detailed in Textbox 1.

**Eligibility criteria.**

**Inclusion criteria based on relevant factors**

- Population
  - Health care and social care professionals
- Intervention
  - Non–knowledge-based clinical decision support systems (CDSSs) [4] deployed for health care
- Comparator
  - Any (including usual care or no comparator)
- Primary outcomes
  - Health care professionals’ experiences of the use of artificial intelligence (AI) tools in health care delivery
  - Benefits and challenges of the use of AI tools in health care
- Secondary outcomes
  - Health care professionals’ understanding of AI tools in health care delivery
  - Perceived and actual impact of AI tools on decision-making and the health system (eg, the need for extra clinicians) and clinical workflow and patient pathway
- Types of study
  - Any primary study (empirical study) of any design (qualitative, quantitative, or mixed methods)
  - Studies that focused on issues relating to health care provision, health outcomes, and health service or system configuration were considered for inclusion
- Context
  - Any health care setting from any country

**Exclusion criteria based on relevant factors**

- Population
  - Nonprofessional caregivers (such as family caregivers)
- Intervention
  - Knowledge-based CDSSs
  - Systems under development or testing
  - AI tools as treatment (eg, robopets)
- Comparator
  - Not applicable
- Primary outcomes
  - We excluded studies which focused on the perception of health care professionals on hypothetical use rather than actual use of AI tools
- Types of study
  - Nonprimary studies (such as literature reviews and opinion papers)
  - Studies exclusively reporting the real-world effectiveness, performance, or diagnostic accuracy were excluded unless accompanied by results relating to health care professionals’ experience relating to the outcomes stated earlier
- Context
  - Studies which are not focused on health care settings

### Data Extraction and Study Quality Assessment

A data extraction form, developed and piloted by the review team, was used to extract relevant information from each article. The following data were extracted from the eligible studies: author names; publication year; study design; study aims and objectives; geographic location; sample size; characteristics of the health care professionals included (including any indication of how long they have been using AI); description of the AI examined; input data (image or tabular or multimodal); platform (eg, electronic medical record based, standalone app or website, or mobile health embedded); tool development (eg, was it developed in house or by a third party?); study funder; and outcomes.

Data were extracted by one reviewer and checked by a second reviewer. Disagreements were resolved by deliberation between the 2 reviewers and, where necessary, a third reviewer or the project team was consulted.

Two authors independently assessed the quality of all included studies using the Mixed Methods Appraisal Tool [14], which was appropriate due to the variety of study designs included in this review. Disagreements were resolved by discussion between the 2 assessors. A third reviewer was consulted to reach a final decision if the independent assessors were unable to reach a consensus.

### Data Analysis

**Figure 1:**
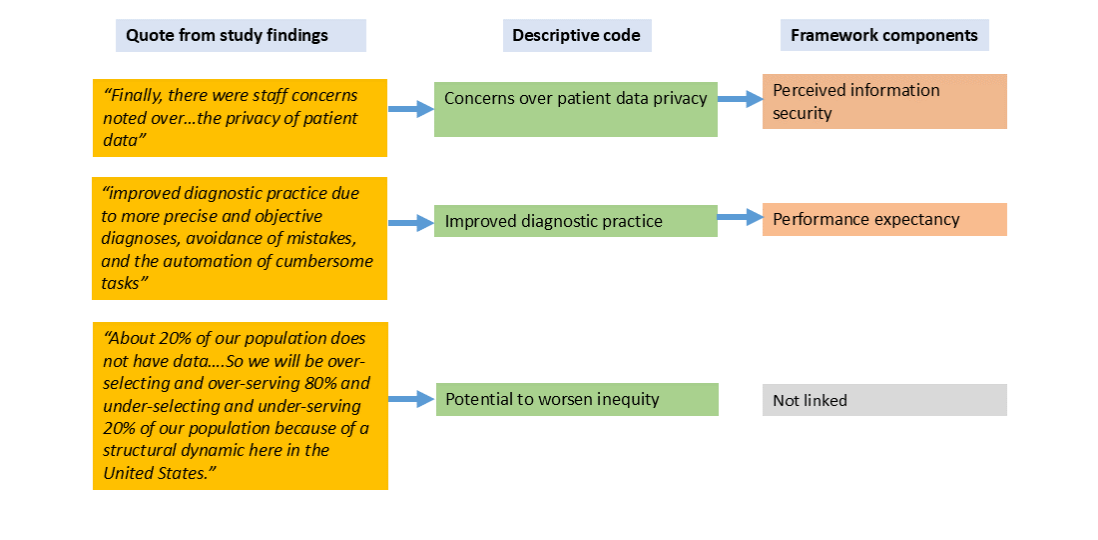
Schematic illustration of the data coding process.

Initially, a theoretically informed thematic synthesis was adopted to analyze the findings of the included studies [15]. The meta-UTAUT was used as a theoretical framework [10,11]. This theoretical framework was selected because it is specific to the acceptance and use of information systems and IT, has been refined through meta-analysis, and is extensively used for understanding adoption of information systems and IT [11]. Two independent reviewers coded the information from the findings of the included studies into descriptive codes and linked these codes to the components of the meta-UTAUT framework (Figure 1). During coding, we remained alert to information that did not fit the framework (Figure 1). We used NVivo software (Lumivero) to facilitate data coding process. Codes and coding were reviewed by the team and modifications discussed. Although the meta-UTAUT allowed us to understand the data, we noticed that the coding was fragmented and the connections between the codes were being missed. Therefore, we decided to describe findings based on the broader themes.

After coding, we made a judgment as to whether codes were specific to AI or not by undertaking a thought experiment supported where necessary by published evidence. Team members (AA and FG) asked themselves, “Could this code be applied to another digital intervention including knowledge-based clinical decision support?” If the answer was yes, we labeled it as “nonspecific.” Examples of nonspecific codes include problems with internet connection, because this will cause a problem with any digital system requiring internet connection (eg, video calls); challenges with using digital systems, because it can surface where insufficient training has been given [16]; alert fatigue, because it is a recognized problem for any CDSS [17]; and the importance of ensuring that systems do not adversely affect workflows, because it is a recognized issue for the design of digital interventions [18]. In the Results section, we provide examples of the nonspecific codes. We identified and focused our analysis on the codes specific to AI tools. Through a process of iteration, discussion, and independent verification, we identified 7 overarching themes that encapsulate the data.

Quotes from the findings of the studies were coded into descriptive codes and linked to the components of the meta-UTAUT [10,11]. In cases where no direct link to the meta-UTAUT components existed, those codes were still retained for analysis.

### Stakeholder Engagement Workshop

We conducted a web-based stakeholder engagement workshop in August 2022 to obtain feedback on our initial findings and recommendations for future research and practice. We invited patient and public representatives, health care professionals, experts in AI, and policy makers. Potential participants were invited through direct personal contacts. We sent out advertisements to National Institute for Health and Care Research Applied Research Collaboration West Midlands patient and public representatives, Young Person’s Mental Health Advisory Group of King’s College London, and Cross-Applied Research Collaboration working group on AI. A 2-hour workshop was held over Zoom (Zoom Video Communications, Inc) where we presented our evidence synthesis and invited attendees to provide feedback on our initial findings and recommendations of any additional research that could be conducted in this area as well as any practice implications arising from the existing findings.

## Results

### Study Selection

**Figure 2:**
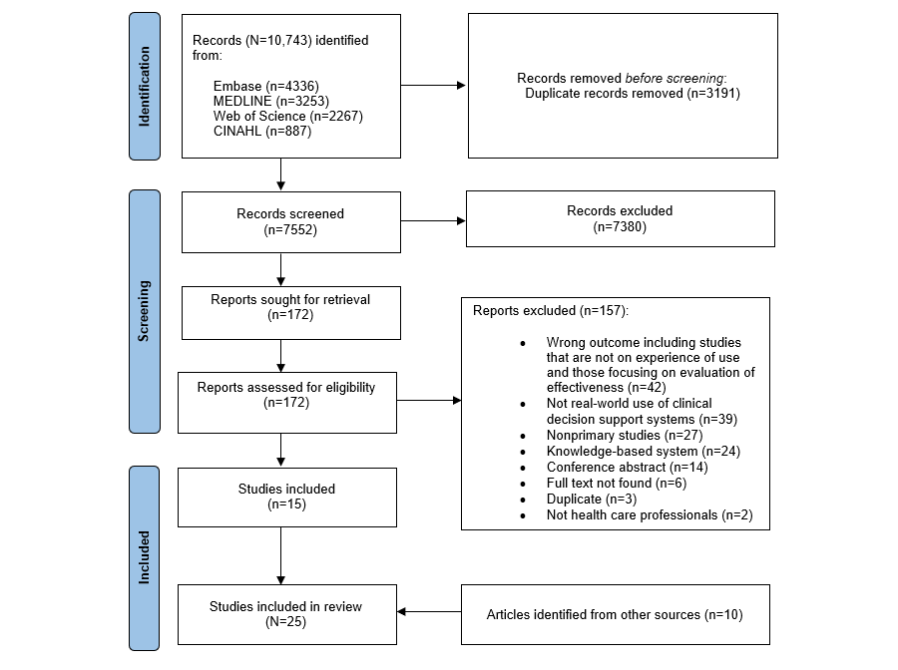
PRISMA (Preferred Reporting Items for Systematic Reviews and Meta-Analyses) flowchart of study selection.

A total of 10,743 records were retrieved from the electronic databases. After the removal of duplicate records and screening based on titles and abstract, 172 full-text articles were screened. An additional 10 studies were identified from other sources. Overall, 25 studies describing the experiences of health care professionals from differing disciplines and levels of seniority with various AI met the selection criteria and were included in the final review. The process of study selection is shown in Figure 2. List of excluded studies and the reasons for exclusion are presented in Multimedia Appendix 2.

### Study Characteristics

The characteristics of the included studies are presented in Multimedia Appendix 2. The included studies (N=25) were published from 1990 to 2022 with 92% (n=23) of the studies published between 2019 and 2023. The studies covered 9 countries, with the majority being reported from the United States (n=12), then the Netherlands (n=2), Canada (n=2), Austria (n=2), and one each from the United Kingdom, Spain, Switzerland, Argentina, Brazil, China, and Thailand. The included studies were primarily qualitative (13/25, 52%), with 36% (9/25) being quantitative and 12% (3/25) being mixed methods studies. Sample sizes ranged from 12 to 724 participants, although 2 studies did not report these data [19,20]. Participants of the included studies comprised practitioners of various disciplines spanning several clinical specialties. The types of AI systems used in the included studies varied widely, covering those used in risk prediction, diagnosis, and treatment (Table 1).

**Table 1:** Description of the AI^a^ systems used in included studies.

Study, year	Study type	Name of AI system	Clinical decision-making task	Setting; country	Target population	Tool development
Beede et al [21], 2020	Qualitative study	No name given	Assessment of diabetic retinopathy	Primary care clinics; Thailand	Patients with diabetes requiring an eye examination	In house
Cruz et al [22], 2019	Quantitative study	Savana system	Notifies physicians in real time about recommendations regarding the health care process to improve adherence rates to clinical pathways	Primary care clinics; Spain	Patients attending primary care	Commercial
Frymoyer et al [23], 2020	Quantitative study	InsightRX-MIPD CDS tool	Determines the optimal dosing regimen of vancomycin	Hospital; United States	Neonates and children for suspected or documented infections with MRSA^b^, methicillin-resistant coagulase-negative Staphylococci, and other drug-resistant gram-positive organisms	Commercial
Ginestra et al [24], 2019	Quantitative study	EWS 2.0 alert	Sepsis risk prediction	Hospital; United States	Non–intensive care unit admissions	In house
Gonçalves et al [19], 2020	Qualitative study	Robot Laura	Early identification of sepsis	Hospital; Brazil	Patients in hospital	Commercial
Henkel et al [25], 2022	Quantitative study	AI-Pathway Companion Prostate Cancer VA10B	Diagnosis and management of prostate adenocarcinoma cancer	Hospital; Switzerland	Patients undergoing prostate cancer screening	Commercial
Henry et al [26], 2022	Qualitative study	TREWS^c^	Sepsis detection and treatment management	Hospital; United States	Patients in hospital	Unclear
Jauk et al [27], 2021	Mixed methods study	Unclear	Predicting delirium	Hospital; Austria	Every patient admitted to one of the departments	Unclear
Jauk et al [28], 2022	Quantitative study	Delirium prediction software	Delirium risk prediction	Hospital; Austria	Patients in hospital	In house
Jordan et al [29], 2022	Qualitative study	KATE	Clinical triage	Hospital; United States	Patients at the emergency department	Commercial
Joshi et al [30], 2022	Qualitative study	No name given	Sepsis risk prediction	Hospital; United States	Patients at risk of sepsis	Commercial and in house
Kappen et al [31], 2016	Mixed methods study	No name given	Predicting the risk of PONV^d^	Hospital; Netherlands	All surgical patients	Unclear
Lebovitz et al [32], 2022	Qualitative	No name given	Diagnosis of breast cancer, lung cancer and estimation of bone age	Hospital; United States	Patients requiring diagnostic radiology	Unclear
Marwaha et al [33], 2021	Qualitative study	Face2Gene	Diagnosis of children with rare genetic syndromes	Hospital; Canada	Children with rare genetic syndromes	Unclear
McAdam et al [20], 1990	Quantitative study	Leeds Abdominal Pain System	Diagnosis of acute abdominal pain	Hospital; United Kingdom	Patients presenting with acute abdominal pain	In house
Nehme et al [34], 2023	Quantitative study	GI Genius	Colorectal polyp detection	Cancer center; United States	Patients undergoing elective outpatient colonoscopy	Commercial
Rabinovich et al [35], 2022	Mixed methods study	TRx	Chest x-ray interpretation	Hospital; Argentina	Patients at the emergency department	In house
Romero-Brufau et al [36], 2020	Quantitative study	No name given	Identify patient at risk for poor glycemic control in the next 3 months	Primary care clinics; United States	Patients at risk of poor glycemic control	Commercial
Sandhu et al [37], 2020	Qualitative study	Sepsis Watch	Sepsis risk prediction	Hospital; United States	Patients in hospital	In house
Saunders et al [38], 2021	Qualitative study	The mHOMR^e^ tool	Mortality risk prediction	Hospital; Canada	Patients with non–cancer serious illnesses	Unclear
Shiang et al [39], 2022	Quantitative study	AI-based decision support system	Detection of pulmonary embolism, intracranial hemorrhage, and acute cervical spine fractures	Hospital; United States	Patients at the radiology department	Commercial
Singer et al [40], 2022	Qualitative study	No name given	Readmission tool: readmission risk prediction	Hospital; United States	All patients admitted to hospital	Unclear
Strohm et al [41], 2020	Qualitative study	BoneXpert	Bone maturity assessments based on x-rays of pediatric patients’ hands	Hospital; Netherlands	Pediatric patients	Commercial
Sun and Medaglia [42], 2019	Qualitative study	Watson for oncology	Personalized cancer management	Hospital; China	Patients with cancer	Commercial
Wang et al [43], 2023	Qualitative study	No name given	Predicting risk of PAD^f^	Community or primary care; United States	Patients with PAD who had at least one visit to Duke Health with a PAD-related diagnosis code between January 2015 and March 2016	In house

^a^AI: artificial intelligence.

^b^MRSA: methicillin-resistant *Staphylococcus aureus*.

^c^TREWS: Targeted Real-Time Early Warning System.

^d^PONV: postoperative nausea and vomiting.

^e^mHOMR: modified Hospitalised-patient One-year Mortality Risk.

^f^PAD: peripheral arterial disease.

### Quality of Included Studies

The quality of included studies is presented in Multimedia Appendix 2. Overall, the quality of most of the included studies was good. Of the 25 included studies, 12 qualitative studies [19,21,26,29,32,33,37,38,40-43] fulfilled all the relevant quality criteria on Mixed Methods Appraisal Tool while 5 studies fulfilled at least 80% of the quality criteria [20,22,24,27,30]. The remaining 8 studies fulfilled ≤67% of the quality criteria [23,25,28,31,34-36,39], and most of the unmet criteria were assessed as “can’t tell” primarily due to the lack of sufficient information.

### Excluded Generic Issues

We identified a range of nonspecific issues related to the implementation of digital technologies in health care (see Textbox 2 for examples). These issues have already been extensively addressed in the existing literature [8] and were therefore excluded from analysis in our study.

**Examples of broader issues relating to implementation reported in included studies.**

- Issues relating to abandonment [30,41]
- Competing priorities, especially in cases where health care professionals felt the artificial intelligence (AI) was not particularly useful [31,38]
- Limited infrastructure to support the adoption of the technology [19,21]
- Health care professionals’ satisfaction with AI applications [19,23,25,30]
- Ease of use [25,27,28,35]
- Integration into the existing systems and accessibility within the existing system [23,27,41]
- Health care professionals appreciated systems that do not affect the dynamics of their workflow [22,27]
- Concerns about alert fatigue or redundancy of alert [30,38]
- Technological challenges relating to health care professionals’ proficiency in the use of hardware and software [19]
- Issues relating to information security, including the necessity of secure transmission of data [23], concerns over privacy of patient data [33], and potential threat to national security [42]
- Lack of complete data for patients [43]
- Lack of standards for what and how data are collected [42]
- Issues with internet connections [19,21]
- Challenges with hardware such as recording equipment, smartphones, and tablets [19]

### Key Themes

#### Overview

In this section, we describe the experiences of health care professionals in using AI tools to support clinical decision-making, as reported in the included studies. We focused on the nuances we believe are specific to AI following the process described in the Methods section. We identified seven themes: (1) understanding of AI applications; (2) level of trust and confidence in AI tools; (3) judging the added value of AI; (4) data availability and limitations of AI; (5) time and competing priorities; (6) concern about governance; and (7) collaboration to facilitate the implementation and use of AI tools. We provide detailed exploration of each theme in subsequent sections.

#### Health Care Professionals’ Understanding of AI Applications

In total, 10 studies reported concerns with health care professionals’ lack of understanding of AI applications. This includes lack of understanding of the outputs of the AI application and the algorithms or the rationale for the outputs by the AI applications due to the lack of transparency relating to non–knowledge-based AI applications [24,26,27,30,32,37,38,40-42]. For example [42],

This lack of transparency is perceived as a major challenge; the AI technology represents as a “black box”, and its users have no power to understand its mechanisms, or modify them to tackle potential problems.

As another example, a study on the use of a machine learning sepsis early warning system reported as follows [37]:

Both RRT [rapid response team] nurses and ED [emergency department] physicians said that they lacked the knowledge and understanding required to assess the validity of the machine learning model...Physicians also lacked knowledge about the model and the predictive nature of the model.

Another study on sepsis risk prediction reported confusion regarding understanding what the alert means [30]. In an attempt to minimize this issue, inclusion of an explanation for the alert firing was introduced in some hospitals. Some felt the explanations were helpful while others found it more confusing [30]:

A lot of people get confused...so say you get 25, when the patient’s really sick and then the number goes to twenty, does that mean the patients getting better? What do all of the subsequent numbers mean? If it goes up to 30, is the patient getting worse?

Concise content with explanations for firing was well-received. A few noted that this was not possible or was more confusing, but most felt inclusion of explanations was helpful.

Participants in a study on the use of AI tools in radiology reported as follows [32]:

Dr V: How does [the AI tool] know that this is a nodule, but this isn’t?

Dr C: They all look identical to me.

Dr K: They expressed frustration in their inability to understand the divergent AI results: What is it telling me to look at? At this tissue? It looks just like the tissue over here, which is perfectly normal...I have no idea what it’s thinking.

However, in a study [26] on an early warning system for sepsis, limited understanding of how the AI system operates was not considered a major barrier to the use of the application.

#### Level of Trust and Confidence in AI Tools

Findings related to trust in the accuracy and judgments or recommendations of AI tools are mixed [19,21,26-28,30-37,39,41,43]. Some health care professionals believe that the AI tool performs as expected while others do not, and this was reported across various AI tools. For example, in a study [30] on a sepsis risk prediction tool, implementers were unsure whether the AI tools were able to predict sepsis with clinically meaningful specificity when compared with the traditional early warning system using a systemic inflammatory response system, which relies on physiological data (vital sign and laboratory abnormalities). Participants noted feeling disappointed about the predictive potential [30]:

The tool...was supposedly predictive, but we discovered...it wasn’t predictive...it was really telling providers that they’ve met the criteria for severe sepsis which...is not really predictive because they’ve already met it. It wasn’t that you were getting it before it happened so even though they were selling it as a predictive model I’m not so convinced it was predictive.

In another study on the use of AI to support delirium prevention, the following was mentioned [27]:

[S]even users (14.9%) did not believe that the application is a useful support for delirium prevention, and seven did not believe that the application can be used to detect delirium at an early stage.

Referring to the use of an AI tool for lung cancer diagnosis, a study reported the following [32]:

Radiologists were deeply committed to providing judgments with maximum certainty, but they expressed difﬁculty feeling certain given the opacity they experienced when considering divergent AI results: “I just don’t know of any radiologist who’s not looking closely at the case because they have AI. Because at the end of the day, you’re still responsible. How can you trust the machine that much?”

Dr E

In a study among health care professionals on the use of BoneXpert, some health care professionals reported struggling to accept outputs from the application [41]:

Interestingly, in three hospitals, we found that the referring clinicians did not trust the output of the AI application and redid a manual bone age analysis for every scan. Thus, just like the radiologists, the referring clinicians showed varying levels of acceptance of AI applications.

Divergence in the opinions of health care professionals and AI recommendations creates uncertainty. However, by using “AI interrogation practices,” radiologists are able to use AI in a way that helps them to experience less uncertainty in making final judgments [32]:

On the surface, it may seem that using the AI tools (and experiencing opacity) increased the overall uncertainty these radiologists experienced; however, in fact, using the AI tool resulted in radiologists experiencing less uncertainty making their ﬁnal judgments. They achieved this by using “AI interrogation practices” or practices that human experts enact to relate their own knowledge claims to the AI knowledge claims.

Other experiences that led to lack of trust in AI tools were the lack of relevant empirical evidence on AI clinical performance and of its impact on health care workflow and quality and the problem for clinicians where AI results do not match their own judgments [32,41]. For example, in a study of the implementation of AI applications in radiology across 7 hospitals in the Netherlands, the authors reported the following [41]:

[T]here is a lack of empirical evidence on the effect of AI applications on the radiological workflow, as well as their added value for clinical radiology practice...measuring clinical and organizational benefits of AI on a microlevel is difficult...publications on the validation of the algorithms are based on laboratory rather than clinical settings.

Societal expectations may also influence confidence in AI tool use. In a study of the use of the Watson tool in China [42], the authors reported that social media and organizations frequently talk about AI and attribute “magic” qualities to AI, which often leads to doctors being disappointed:

Hospital managers or doctors report to experiencing frustration when facing the real technology after the societal hype.

In one study [43], participants suggested that having a track record of the actions of health care professionals based on AI recommendations and the corresponding patient outcomes could facilitate confidence in AI tools.

#### Judging the Added Value of AI

In some studies, health care professionals reported that AI tools provided added value and improved decision-making [19,20,24,27-30,35,36,39-41]. This included automation of burdensome tasks [41], avoidance of mistakes [32,41], reduction in variability in clinical practice [22], improvement in team communication [24,36], reduction of alert fatigue due to the combination of multiple data points [30], improvement in data collection [20] and team performance [19,27], and provision of more objective and precise diagnosis or prediction [41].

In some studies, some health care professionals found that AI tools served as a reminder or confirmation of their clinical judgments [23,26,27,29,32,35,38,39,42]. For example [27],

The prediction helps to corroborate my own estimation when seeing a patient.

However, not all users of the same tool found it useful [27]:

Opinions about the application’s usefulness for their own work were mixed: 17 users (36.2%) reported the application to be useful for their work, while 15 users (31.9%) did not find it useful.

Health care professionals reported some problems relating to AI technologies not addressing issues that are deemed important to them. For example, health care professionals thought postoperative nausea and vomiting (PONV) has low burden and should not be excessively treated [31]. In a study in a US hospital among health care professionals using AI tools for diagnosing breast cancer, lung cancer, and bone age determinations [32], radiologists said AI tools identify abnormalities that they might not see but which were not clinically significant to impact their final judgment:

Calciﬁcations can be really little and sometimes hard to see. It [Mammo AI tool] sees those calciﬁcations better than I do. But it also sees all kinds of calciﬁcations that are neither here nor there.

Dr B

In a study of peripheral arterial disease (PAD) identification algorithm [43], clinicians emphasized the importance of ensuring that the AI tools focus on supporting clinical decision-making by performing additional analysis that the clinicians do not normally do:

I mean, it [the algorithm] has to solve a real problem. Like, I’m not interested in models that in a clinical sense, identify data that I could just identify in the course of my daily work.

Some studies also reported that health care professionals expressed uncertainty about next steps and that AI tools provided no actionable outputs, therefore not adding value to their clinical decision-making [31,38,43]; adding treatment recommendations would have been more useful [31]. For example, referring to a tool to predict the risks of PONV, a physician said the following [31]:

The intuitive use of the predicted PONV [postoperative nausea and vomiting] risk and a stated preference for an actionable recommendation by intervention group interviewees suggested that being presented only with a predicted risk may be difﬁcult to use in a clinical decision. Adding a risk-corresponding treatment recommendation may assist physicians in interpreting the predicted risk for a decision on PONV prophylaxis.

Theme 4G, quote 43

Health care professionals also voiced concerns over AI tools not providing actionable information [38]:

It would have been nice to have some sort of actionable items, because while the information is good to know, I was never really sure what to do with it. It’s like, great, my patient has an elevated one year mortality risk. What can I do about that? What do I do with this information?

RES02

From another study [40]:

They gave us the risk scores, but we did not really understand what to do with this information, what do these scores mean? It took us a while to figure out what is our threshold for what are we going to consider high risk patients.

Readmission Risk Tool user

Furthermore, in a study of an automated mortality prediction model for identification of patients for palliative care, AI helped reduce uncertainties, especially in less-experienced health care professionals [38]. In some situations, AI tools reportedly caused added work for health care professionals [21,29,34,37,41]. For example, in a study on the use of AI for the detection of diabetic retinopathy [21], the AI tool required high quality data and or images, which may be difficult to achieve in some cases. A study participant reported the following:

It gives guaranteed results, but it has some limitations. Some images are blurry, and I can still read it, but [the system] can’t. P3 shared the same sentiment, “It’s good but I think it’s not as accurate. If [the eye] is a little obscured, it can’t grade it.” The system’s high standards for image quality is at odds with the consistency and quality of images that the nurses were routinely capturing under the constraints of the clinic, and this mismatch caused frustration and added work.

In one of the studies on the implementation of AI in diagnostic radiology [41], the clinical benefits or organizational goals for using AI applications were not clearly established before implementation, making it hard to assess after implementation:

From an organizational perspective, clinical benefits or organizational goals that might be achieved by using AI applications are not clearly established ex-ante and therefore hard to assess after implementation.

Health care professionals were concerned that patients may not be willing to have AI tools used for them [42]:

They [the patients] have no idea about Watson [a system for designing personalized treatment for cancer patients]. They will think: why do I need a machine to look at [my problem]? I prefer an expert doctor.

Health care professionals expressed concerns about limiting treatment for palliative care patients based on the risk prediction by AI tools [38]. There were also concerns about clinicians and regulatory agencies becoming overly reliant on AI [26,29]. For example, health care professionals may not actively consider their cases and they may not be able to refine their skills and maintain cultural competence [29].

#### Data Availability and Limitations of AI

A study reported the users’ concern about the potential for AI to worsen inequity as disadvantaged populations are not represented in health datasets [43]:

About 20% of our population does not have data.... So we will be over-selecting and over-serving 80% and under-selecting and under-serving 20% of our population because of a structural dynamic here in the United States.

And I should also say those even worse, those 20% [who we don’t have data on], or a higher proportion of them are older, coming from an economically higher social deprivation index, higher disease burden, nonwhite.

Concerns about the limits of AI in considering all the aspects of patients’ lives were also talked about [31,43]. For example, in the study [31] on an AI tool for PONV, the following was discussed:

[T]hey [physicians] felt that a prediction model does not take into account all aspects of a speciﬁc patient. The prediction model only predicted the risk for a speciﬁc outcome and did not weigh the beneﬁt of treatment against the expected harms and contraindications for a particular patient with speciﬁc characteristics and comorbidity.

In the study of the use of PAD identification algorithm [43], health care professionals reported having access to more data about patient than AI algorithm uses. There were concerns about the AI tool not being able to account for factors such as patient’s culture [29] and ground context and therefore limiting the potential impact of the AI [43]:

Exercise, nutrition, smoking, and medication adherence are critical components of PAD [peripheral arterial disease] management and clinicians expressed concern about adopting the algorithm to make intervention recommendations without knowing more about these factors...The algorithm’s potential impact was limited by an inability to account for barriers patients faced in managing PAD.

#### Time and Competing Priorities

Health care professionals reported lack of time to fully utilize AI tools. For example, in a study on the use of the modified Hospitalised-patient One-year Mortality Risk tool, physicians reported lacking time to address the alert because they were often focused on the acute needs of the patient [38]:

The inpatient stay often is very compressed. They’re in the hospital, they’re getting treated, and then they’re home. And so, there is not time during the inpatient stay to address these things.

In the study of the use of AI for PAD identification [43], health care professionals reported limited time to review high-risk patients and having difficulties in choosing which patients to prioritize:

The PAD [peripheral arterial disease] ML-driven CDS can be run on all patients in a population, but only a small number of high-risk patients could be reviewed each week. Stakeholders described the difficult trade-offs they had to make when considering how to pick patients to prioritize for Population Rounding.

Time constraint could be further heightened where AI recommendations differ from the clinical judgment of the health care professionals [32]. However, in a study on the use of AI in diagnostic radiology, radiologists were willing to invest additional time to relate their own knowledge to the AI knowledge claims to build an understanding of the AI results and reconcile divergent views [32]:

I know my limitations and I know this [CT AI] is going to help them [nodules] stand out a little better. It’s worth the extra time in my mind.

#### Concern About Governance

Health care professionals expressed concerns over regulatory and legal uncertainties surrounding AI use [41,42]. For example, uncertainties regarding legal responsibilities for misdiagnosis stemming from AI recommendations were reported in a study in the Netherlands [41], and this may vary for different countries. In another study [42], a health care professionals reported that it is illegal for AI applications to make clinical decisions in China:

In China it is illegal for an AI system to make a decision.

1HP05

In our hospital, we use Watson to assist the Multidisciplinary Team (MDT).... We discussed how to use Watson for a long time.... As the first hospital to use Watson, we find this way [to use Watson together with the MDT].... But this really gives us a heavy burden! Because when we use Watson, we must have at least five doctors to work together with Watson [as required by regulation]. They [the 5 doctors] will sign on the report.

1HP05

#### Collaboration to Facilitate the Implementation and Use of AI

In a study [40] on the use of low bed tool and readmission risk tool, the authors described how health care organizations can enable collaboration between key stakeholders (users, developers, and outside experts) to facilitate the development and implementation of AI. This collaboration allowed users (care management and utilization management team members) to be involved in the identification of initial needs, identification of new users, need for experts, identification of potential sources of inaccuracy, and possible areas of improvement [40].

Having health care professionals serve as champions or leads for AI use and having knowledge exchange platforms supported the use of AI [26,41,43]. For example, the study [41] on the implementation of AI applications in clinical radiology reported that local champions (radiologists with keen interest in AI and good understanding of AI applications) are vital in encouraging implementation within their departments. In another study [43], having a physician lead the integration of AI tool for PAD identification was reported to be beneficial in facilitating the translation to create real-world clinical impact:

Dr. XXX obviously was very aware, she was kind of the one that spearheaded [the integration] and wanted to get this going. So I think you know, [targeting clinical need] is her role. I’m sure with her specialty, she probably realized that it was an area that needed more attention.

Operational stakeholder

Furthermore, in the study of the use of AI for early warning for sepsis [37], a new role of a sepsis watch nurse was created. The sepsis watch nurses were the primary users of the tool and were extensively trained in the workflow and the tool. They were responsible for communicating the outputs from the AI tool to physicians to facilitate the translation of the recommendations to patient’s bedside. With regard to the skills required to fulfill such roles, the nurses recommended the following [37]:

When asked about the skills and knowledge needed to be a good Sepsis Watch nurse, the RRT nurses mentioned good clinical judgment, knowledge of sepsis, and critical care experience...RRT nurses also explained the importance of strong communication skills to confidently speak with attending physicians whom they may not personally know...RRT nurses thought that strong computer skills were not necessary for the role, given the simplicity of the app. RRT nurses also recommended recruiting nurses interested in the role and the need to create buy-in through continuous feedback.

In another study [26] on the early warning system for sepsis, the deployment team attended staff meetings and also met with individual users, as requested, to explain the system and provide guidance on using the interface. Users were also able to ask questions about the system’s behavior using a feedback button [26].

In a study [41] on the implementation of AI tools in diagnostic radiology, it was reported that only 1 of 7 hospitals had a formalized innovation strategy regarding AI, although 3 hospitals had a designated innovation manager. It is worth noting that 4 more hospitals were reported to be developing a structured approach at the time of the study. The lack of structured implementation processes lead to substantial variations in how the application is used in different departments [41]:

From a workflow perspective, implementation plans do not specify how the AI application should be integrated into the clinical workflow, which leads to significant variations in the way the application is used in different departments. Furthermore, in all cases, the work done to monitor existing practices or the impact of the implementation of novel technologies on the level of the hospital is currently limited.

### Key Points From Stakeholder Engagement

A total of 18 individuals attended the stakeholder engagement workshop, besides the study team and 1 patient and public engagement officer. This included 9 patient and public representatives, 7 researchers from diverse related fields (including clinical researchers), 1 representative from an AI technology company, and 1 policy maker in data and AI. After being presented with the study findings, the participants agreed that algorithmic transparency and an understanding of how AI makes predictions may be challenging with non–knowledge-based AI. They believed that health care professionals are aware that AI will be used more in the future, but they do not feel prepared for it. The participants highlighted the need for more evidence to showcase good practice and collaborations across specialties or disciplines. They recommended early training of health care professionals to improve their understanding of what AI is and how it works. They acknowledged that some of the training programs may be based on theory rather than wait until AI is fully deployed in all clinical settings. They also recommended the need for AI applications guidelines to include examples of how conflicts between AI recommendations and health care professionals’ judgments should be resolved. With regard to suggestions for future research, they suggested that more case studies and mixed methods research would be useful to provide insights into human perspectives of the quantitative research and explore health care professionals’ confidence in AI systems. Participants recommended baseline studies on clinicians’ knowledge and experiences of AI systems to provide comparison data for future work. There were also suggestions of a need for more information on the perspectives of hospital management and patients on the real-world use of AI systems. Differences in perspectives based on individual characteristics (such as age) could also be explored.

## Discussion

### Principal Findings

This review describes existing empirical evidence on health care professionals’ real-world experiences of using non–knowledge-based CDSSs. While some of the issues emphasized in the included studies can be attributed to issues in implementation of any technology or intervention, we identified many issues that appeared to be particularly challenging for non–knowledge-based CDSSs. We grouped these issues into seven themes: (1) health care professionals’ understanding of AI applications; (2) level of trust and confidence in AI tools; (3) judging the added value of AI; (4) data availability and limitations of AI; (5) time and competing priorities; (6) concern about governance; and (7) collaboration to facilitate the implementation and use of AI. The most frequently occurring issues included concerns over lack of understanding of AI outputs and the algorithm or rationale for the AI output. In addition, many studies highlighted the issue of trust and confidence in AI tools where some health care professionals believe that the AI tools function as anticipated while others hold contrary opinions. This lack of trust is further compounded when there are divergent opinions of health care professionals and AI recommendations. Another notable issue relates to the added value of AI. Some health care professionals reported that AI added value whereas some believed it did not add value, especially when the AI technologies did not address issues that were important to the health care professionals or failed to produce any actionable outputs. The challenges are interlinked; lack of understanding of the AI affects the ability to use the output and so trust cannot develop and health care professionals struggle to see the value in the AI tools and remain concerned about clinical responsibility and liability. The regulatory context and how the AI tool was implemented influence their approach to using it.

### Comparison With Other Work

Other authors have found that the ability of health care professionals to trust non–knowledge-based CDSSs and understand how they work to benefit patients or improve care delivery are important factors in their adoption from the perspectives of both health care professionals and the public [44-46]. The lack of robust empirical evidence to support the use of these systems in health care is thought to contribute to these issues [47,48]. One study used the expectancy-value theory [49] and modified extended Unified Theory of Acceptance and Use of Technology [50] to understand how expectancy (effort expectancy and performance expectancy), trust, and perceptions of clinicians related to their intention of using an AI-based CDSS: the Blood Utilisation Calculator [51]. The findings showed that expectancy and perceived risk on using the application have a substantial effect on trust [51]. Establishing a foundation of trust is crucial for the acceptance and effective integration of various AI technologies into health care practices [52].

It has been argued that for machine learning or AI systems to become trusted and accepted in health care, clinicians, researchers, and patients need to feel that the conclusions the systems make and the way that they reach them are interpretable and explainable [53]. This includes the clinician understanding the limitations of the system as a result of limitations of data quality [54] and the complexity of the data [55]. However, studies suggest that whether explainability does add value to AI powered clinical decision support depends on the context it is being used and by whom in addition to technical considerations [56,57]. In contrast, it has been argued that accuracy is sufficient for day-to-day clinical practice, with explainability being a research endeavor [58].

The view that algorithmic bias can exacerbate inequalities is supported by empirical evidence [59]. These biases can be mitigated to some extent [60]. Regulators recommend that the data used in training algorithms be representative of the intended patient population, and an international initiative is underway to develop recommendations for the composition and reporting of datasets used in AI systems for clinical practice [61]. However, as our findings indicate, health care professionals may have data about their patient that is missing from the datasets used in diagnostic CDSSs [4].

The inability of clinicians to process all the information presented to them is not new but potentially worsened by digitally available information, particularly in time-pressured clinical settings [62]. The need for health care organizations to optimize CDSS alerts is not limited to non–knowledge-based systems [63]. The concern expressed about clinical responsibility and liability for non–knowledge-based CDSSs is widely shared [64]. Regulation is in the works, but solutions are not straightforward as there is interaction between AI trustworthiness and transparency and how the AI tool and clinicians work together, which also changes the clinician-patient interaction [64,65]. An extensive systematic review of the barriers and enablers to the implementation of CDSSs for chronic disease identified similar implementation issues as in our review, such as the importance of a champion for the system, the deployment of allocated personnel to use the system, and the need for accessible training [66]. A recent study explored the experiences of various stakeholders, including health care professionals and regulatory bodies in developing and using AI technologies [67]. The study’s recommendations for promoting the deployment of AI align with our findings and those identified in the stakeholder engagement workshop. These shared recommendations emphasize the importance of establishing guidelines for AI technology development and adoption; enhancing cocreation; and providing comprehensive education and training for health care professionals, patients, and communities [67].

### Future Directions

Many studies have examined the perceptions of health care professionals on the use of AI in the health care settings [46,68]. However, the majority were not focused on AI applications that are fully deployed in real-world settings, and even fewer have focused on non–knowledge-based CDSSs. This review provides a more nuanced understanding of the challenges faced by health care professionals in contexts where non–knowledge-based systems are fully deployed. Many of the challenges identified in our review could be mitigated by collaboration with various stakeholders, including health care professionals, patients, and regulators, during the design and development stage of AI systems [69]. By involving key stakeholders in the process, developers gain invaluable insights into the practical needs and concerns of health care professionals, facilitating the creation of AI tools that are more aligned with the real-world requirements. Regular evaluation and validation of AI tools are essential to generate necessary data that are important for end users [70]. This will provide empirical evidence of the utility of the tools, clinical effectiveness, and generalizability [70]. Furthermore, the provision of education and training is necessary as this would equip health care professionals with the necessary knowledge and skills to enhance their confidence and competence in using AI in their practice [67]. With these issues solved as AI development matures, further research on the experience of health care professionals using AI can focus on identifying unintended consequences of AI use in routine clinical practice.

### Limitations

The methods used in this systematic review were established a priori. We used a comprehensive search strategy, searched 4 electronic databases, and contacted experts to ensure we identify relevant studies. Due to limited time and human resources, we were unable to perform independent double screening at the title and abstract screening phase. However, at least 20% of all identified articles were randomly screened by a second reviewer and full-text articles were independently screened by 2 reviewers. Although we dedicated substantial effort to creating a comprehensive search strategy, numerous included studies were identified through recommendations rather than the structured search. This underscores the inherent challenge in rigorously searching databases for this topic, given the diverse language used to describe CDSSs. Moreover, it was difficult to judge sometimes whether the AI tool discussed in a study is knowledge based or non–knowledge based, particularly due to limited descriptions in some studies. Consequently, we recognize the possibility of overlooking some potentially relevant articles. Despite this limitation, our findings align with existing studies, and we believe that this review effectively captures the crucial aspects of health care professionals’ experiences in this domain.

### Conclusions

This review emphasizes the complex challenges faced by health care professionals using non–knowledge-based CDSSs. Issues related to health care professionals’ understanding AI applications, trust in AI tools, and assessing added value are prominent, and the interlinked nature of the challenges identified is evident. As we navigate the evolving landscape of health care technologies, it is important to acknowledge and address these challenges through targeted strategies to improve collaborative development, continuous evaluation or validation, education, and refining the regulatory framework. This will potentially enhance the acceptance and use of AI within health care settings.

## Appendix

Multimedia Appendix 1 Search strategy for all databases.

Multimedia Appendix 2 Data extraction, quality assessment, and list of excluded studies.

Multimedia Appendix 3 PRISMA checklist.

## Abbreviations

AI: artificial intelligence
CDSS: clinical decision support system
meta-UTAUT: meta-analysis–based Unified Theory of Acceptance and Use of Technology
PAD: peripheral arterial disease
PONV: postoperative nausea and vomiting
PRISMA: Preferred Reporting Items for Systematic Reviews and Meta-Analyses
PRISMA-AI: Preferred Reporting Items for Systematic Reviews and Meta-Analyses Artificial Intelligence

## Data Availability

All data generated or analyzed during this study are included in this published paper (and its supplementary information files).
