# Supplementary material for: Health Care Professionals’ Experience of Using AI: Systematic Review With Narrative Synthesis
Source: J Med Internet Res. 2024 Oct 30;26:e55766. doi: 10.2196/55766 (PMC11561443; doi:10.2196/55766)
Supplement: Multimedia Appendix 1 [file jmir_v26i1e55766_app1.docx]

**Multimedia Appendix 1: Search strategies for electronic databases**

Ovid MEDLINE(R) ALL <1946 to June 22, 2023>

| **#** | **Query** | **Results from 23 Jun 2023** |
| --- | --- | --- |
| 1 | ("Health Personnel" or "Healthcare Professionals" or "Health Care Professionals" or doctor* or nurse* or physician* or therapist* or pharmacist* or clinician* or practitioner* or Anaesthetist* or Audiologist* or physiologist* or cardiologist* or immunologist* or microbiologist* or dentist* or dietitian* or paramedic* or Gynaecologist* or midwives or midwife or obstetrician* or Ophthalmologist* or Optometrist* or Orthoptist* or Orthotist* or Paediatrician* or Pathologist* or Phlebotomist* or Physiotherapist* or Podiatrist* or chiropodist* or Prosthetist* or Psychiatrist* or Psychologist* or Psychotherapist* or Radiographer* or Radiologist* or surgeon*).mp. [mp=title, book title, abstract, original title, name of substance word, subject heading word, floating sub-heading word, keyword heading word, organism supplementary concept word, protocol supplementary concept word, rare disease supplementary concept word, unique identifier, synonyms, population supplementary concept word, anatomy supplementary concept word] | 2,182,113 |
| 2 | exp Health Personnel/ | 611,363 |
| 3 | ("home care" or "care home*" or "nursing home*" or "home* for the aged" or "community health services" or "nursing care" or "point of care").mp. [mp=title, book title, abstract, original title, name of substance word, subject heading word, floating sub-heading word, keyword heading word, organism supplementary concept word, protocol supplementary concept word, rare disease supplementary concept word, unique identifier, synonyms, population supplementary concept word, anatomy supplementary concept word] | 236,812 |
| 4 | hospital.mp. or Hospitals/ | 1,542,858 |
| 5 | 1 or 2 or 3 or 4 | 3,629,446 |
| 6 | artificial intelligence.mp. or exp Artificial Intelligence/ | 190,074 |
| 7 | machine learning.mp. or exp Machine Learning/ | 114,684 |
| 8 | deep learning.mp. or exp Deep Learning/ | 49,754 |
| 9 | natural language processing.mp. or exp Natural Language Processing/ | 10,605 |
| 10 | (Boltzmann machine* or Long short-term memory or Gated recurrent unit or Rectified linear unit or Autoencoder or Backpropagation or Multilayer perceptron or Convnet or Support vector machine or Random forest or Lasso or Kernel or Elastic net* or Bayesian or Naive bayes or Genetic algorithm).ab,kf,kw,ti. | 152,668 |
| 11 | ((deep or convolutional or bayesian or neural or elastic) adj1 net*).ab,kf,kw,ti. | 106,765 |
| 12 | ((machine or deep or reinforcement or ensemble or convolutional) adj1 learning).ab,kf,kw,ti. | 133,646 |
| 13 | 6 or 7 or 8 or 9 or 10 or 11 or 12 | 403,852 |
| 14 | ((algorithm* or computeri* or computer-based or computer based or machine-based or machine based or Computer assisted or Computer-assisted or Computer aided or Computer-aided or integrat* or technolog* or digital or electron*) adj3 (decision support or decision-support or decision aid or decision-aid)).ab,kf,kw,ti. | 3,526 |
| 15 | exp Diagnosis, Computer-Assisted/ | 86,738 |
| 16 | computer assisted.mp. | 352,821 |
| 17 | exp Surgery, Computer-Assisted/ | 37,851 |
| 18 | computer aided.mp. | 39,291 |
| 19 | Decision Support Systems, Clinical/ or decision support system*.mp. or Decision Making, Computer-Assisted/ | 18,100 |
| 20 | decision making.mp. or Decision Making/ | 275,058 |
| 21 | 14 or 15 or 16 or 17 or 18 or 19 or 20 | 676,989 |
| 22 | 13 and 21 | 62,931 |
| 23 | (experience* or view or views or opinion* or accept* or attitude*).mp. [mp=title, book title, abstract, original title, name of substance word, subject heading word, floating sub-heading word, keyword heading word, organism supplementary concept word, protocol supplementary concept word, rare disease supplementary concept word, unique identifier, synonyms, population supplementary concept word, anatomy supplementary concept word] | 2,696,579 |
| 24 | 5 and 22 and 23 | 3,253 |

**Embase Classic+Embase <1947 to 2023 June 21> Searched 23/06/2023**

1 ("Health Personnel" or "Healthcare Professionals" or "Health Care Professionals" or doctor* or nurse* or physician* or therapist or pharmacist* or clinician* or practitioners or Anaesthetist* or Audiologist* or physiologist* or cardiologist* or immunologist* or microbiologist* or dentist* or dietitian* or paramedic* or Gynaecologist* or midwives or midwife or surgeon or obstetrician* or Ophthalmologist* or Optometrist* or Orthoptist* or Orthotist* or Paediatrician* or Pathologist* or Phlebotomist* or Physiotherapist* or Podiatrist* or chiropodist* or Prosthetist* or Psychiatrist* or Psychologist* or Psychotherapist* or Radiographer* or Radiologist* or surgeon*).mp. [mp=title, abstract, heading word, drug trade name, original title, device manufacturer, drug manufacturer, device trade name, keyword heading word, floating subheading word, candidate term word] 3108468

2 Health Personnel.mp. or exp health care personnel/ 2100906

3 ("home care" or "care home*" or "nursing home*" or "home* for the aged" or "community health services" or "nursing care" or "point of care").mp. [mp=title, abstract, heading word, drug trade name, original title, device manufacturer, drug manufacturer, device trade name, keyword heading word, floating subheading word, candidate term word] 277955

4 hospital/ or hospital.mp. 2886012

5 1 or 2 or 3 or 4 6030534

6 artificial intelligence.mp. or exp artificial intelligence/ 91368

7 machine learning.mp. or exp machine learning/ 424354

8 deep learning.mp. or exp deep learning/ 62605

9 exp natural language processing/ or natural language processing.mp. 12532

10 (Boltzmann machine* or Long short-term memory or Gated recurrent unit or Rectified linear unit or Autoencoder or Backpropagation or Multilayer perceptron or Convnet or Support vector machine or Random forest or Lasso or Kernel or Elastic net* or Bayesian or Naive bayes or Genetic algorithm).ab,kf,kw,ti. 181956

11 ((deep or convolutional or bayesian or neural or elastic) adj1 net*).ab,kf,kw,ti. 131705

12 ((machine or deep or reinforcement or ensemble or convolutional) adj1 learning).ab,kf,kw,ti. 163613

13 6 or 7 or 8 or 9 or 10 or 11 or 12 604891

14 ((algorithm* or computeri* or computer-based or computer based or machine-based or machine based or Computer assisted or Computer-assisted or Computer aided or Computer-aided or integrat* or technolog* or digital or electron*) adj3 (decision support or decision-support or decision aid or decision-aid)).ab,kf,kw,ti. 4813

15 exp computer assisted diagnosis/ or exp computer assisted surgery/ or computer assisted.mp. 1503626

16 computer aided.mp. 49859

17 exp decision support system/ or exp clinical decision support system/ or decision support system*.mp. or exp decision making/ 505083

18 decision making.mp. or exp decision making/ 564407

19 14 or 15 or 16 or 17 or 18 2103923

20 13 and 19 77778

21 (experience* or view or views or opinion* or accept* or attitude*).mp. [mp=title, abstract, heading word, drug trade name, original title, device manufacturer, drug manufacturer, device trade name, keyword heading word, floating subheading word, candidate term word] 3811604

22 5 and 20 and 21 4336

**CINHAL Search 22/06/2023**

| **Search Terms** | **Actions** |  |
| --- | --- | --- |
| S84 | S30 AND S37 AND S83 | (887) |
| S83 | S38 OR S39 OR S40 OR S41 OR S42 OR S43 OR S44 OR S45 OR S46 OR S47 OR S48 OR S49 OR S50 OR S51 OR S52 OR S53 OR S54 OR S55 OR S56 OR S57 OR S58 OR S59 OR S60 OR S61 OR S62 OR S63 OR S64 OR S65 OR S66 OR S67 OR S68 OR S69 OR S70 OR S71 OR S72 OR S73 OR S74 OR S75 OR S76 OR S77 OR S78 OR S79 OR S80 OR S81 OR S82 | (2,299,476) |
| S82 | "point of care" | (12,387) |
| S81 | (MH "Nursing Care+") OR "nursing care" | (316,663) |
| S80 | (MH "Community Health Services+") OR "community health services" | (480,193) |
| S79 | "home* for the aged" | (321) |
| S78 | (MH "Nursing Homes+") OR "nursing home" | (49,704) |
| S77 | (MH "Home Health Care+") OR "home care" | (62,167) |
| S76 | "care home" | (3,608) |
| S75 | "hospital*" OR (MH "Hospitals+") | (662,845) |
| S74 | (MH "Radiologists") OR "Radiologist*" | (23,231) |
| S73 | ""Radiographer*"" | (5,792) |
| S72 | (MH "Psychotherapists+") OR "Psychotherapist*" | (6,445) |
| S71 | (MH "Psychologists") OR "Psychologist*" | (12,514) |
| S70 | "Psychiatrist*" OR (MH "Psychiatrists") | (11,507) |
| S69 | "Prosthetist*" | (452) |
| S68 | "chiropodist*" | (2,094) |
| S67 | (MH "Podiatrists") OR "Podiatrist*" | (4,158) |
| S66 | (MH "Phlebotomists") OR "Phlebotomist*" | (1,079) |
| S65 | "Pathologist*" | (18,662) |
| S64 | (MH "Pediatricians") OR "Paediatrician*" | (15,595) |
| S63 | "Orthoptist*" | (50) |
| S62 | (MH "Optometrists") OR "Optometrist*" | (2,872) |
| S61 | (MH "Ophthalmologists") OR "Ophthalmologist*" | (4,333) |
| S60 | "obstetrician*" | (7,931) |
| S59 | (MH "Surgeons") OR "surgeon*" | (70,625) |
| S58 | "midwife" | (15,964) |
| S57 | (MH "Midwives+") OR "midwives" | (37,348) |
| S56 | "Gynaecologist*" | (4,275) |
| S55 | "paramedic*" OR (MH "Emergency Medical Technicians") | (15,820) |
| S54 | (MH "Dietitians") OR "dietitian*" | (10,108) |
| S53 | "dentist*" OR (MH "Dentists+") | (45,303) |
| S52 | "microbiologist*" | (474) |
| S51 | "immunologist*" | (888) |
| S50 | "cardiologist*" OR (MH "Cardiologists") | (5,246) |
| S49 | "physiologist*" | (535) |
| S48 | (MH "Audiologists") OR "Audiologist*" | (5,425) |
| S47 | "Anaesthetist*" | (4,046) |
| S46 | "practitioner*" | (122,835) |
| S45 | "clinician*" | (129,361) |
| S44 | (MH "Pharmacists") OR "pharmacist*" | (29,871) |
| S43 | "therapist*" | (71,432) |
| S42 | nurse* | (561,940) |
| S41 | (MH "Physicians+") OR "doctor*" | (192,098) |
| S40 | "Health Care Professional*" | (20,360) |
| S39 | "Healthcare Professional*" | (23,878) |
| S38 | (MH "Health Personnel+") OR "Health Personnel" | (651,364) |
| S37 | S31 OR S32 OR S33 OR S34 OR S35 OR S36 | (1,066,560) |
| S36 | attitude* | (416,897) |
| S35 | accept* | (135,127) |
| S34 | opinion* | (51,027) |
| S33 | "views" | (51,895) |
| S32 | "view" | (67,458) |
| S31 | experience* | (555,062) |
| S30 | S23 AND S29 | (12,929) |
| S29 | S24 OR S25 OR S26 OR S27 OR S28 | (421,585) |
| S28 | (MH "Decision Making+") OR "decision making" | (189,378) |
| S27 | (MH "Decision Support Systems, Clinical") OR "decision support system*" | (8,190) |
| S26 | (algorithm* or computeri* or computer-based or computer based or machine-based or machine based or Computer assisted or Computer-assisted or Computer aided or Computer-aided or integrat* or technolog* or digital or electron*) N4 (decision support or decision-support or decision aid or decision-aid) | (5,382) |
| S25 | "computer aided" | (7,290) |
| S24 | (MH "Surgery, Computer-Assisted+") OR (MH "Therapy, Computer Assisted+") OR (MH "Radiographic Image Interpretation, Computer-Assisted+") OR (MH "Image Processing, Computer Assisted+") OR (MH "Drug Therapy, Computer Assisted") OR (MH "Image Interpretation, Computer Assisted+") OR (MH "Radiotherapy, Computer-Assisted+") OR (MH "Diagnosis, Computer Assisted+") OR (MH "Decision Making, Computer Assisted+") OR "Computer-Assisted" | (230,302) |
| S23 | S1 OR S2 OR S3 OR S4 OR S5 OR S6 OR S7 OR S8 OR S9 OR S10 OR S11 OR S12 OR S13 OR S14 OR S15 OR S16 OR S17 OR S18 OR S19 OR S20 OR S21 OR S22 | (60,399) |
| S22 | (machine or deep or reinforcement or ensemble or convolutional) N2 (learning) | (19,504) |
| S21 | "Genetic algorithm" | (406) |
| S20 | "Naive bayes" | (216) |
| S19 | "Bayesian" | (9,146) |
| S18 | "Elastic net*" | (364) |
| S17 | "Kernel" | (1,275) |
| S16 | "Lasso" | (2,134) |
| S15 | (MH "Random Forest") OR "Random forest" | (2,726) |
| S14 | (MH "Support Vector Machine") OR "Support vector machine" | (2,367) |
| S13 | "Convnet" | (3) |
| S12 | "Multilayer perceptron" | (232) |
| S11 | "Backpropagation" | (77) |
| S10 | "Autoencoder" | (92) |
| S9 | "Rectified linear unit" | (6) |
| S8 | "Gated recurrent unit" | (33) |
| S7 | "Long short-term memory" | (277) |
| S6 | "Boltzmann machine*" | (15) |
| S5 | (MH "Natural Language Processing") OR "natural language processing" | (3,663) |
| S4 | (MH "Deep Learning") OR "deep learning" | (10,609) |
| S3 | (MH "Machine Learning+") OR "machine learning" | (19,511) |
| S2 | (deep or convolutional or bayesian or neural or elastic) N2 (net*) | (10,376) |
| S1 | (MH "Artificial Intelligence+") OR "artificial intelligence" | (32,203) |

**Web of Science**

**Web of Science 22/06/2023**

| # | Search Query | Results |
| --- | --- | --- |
| 1 | (TS=((deep or convolutional or bayesian or neural or elastic) NEAR/2 net* )) | 675110 |
| 2 | TS=((machine or deep or reinforcement or ensemble or convolutional) Near/2 learning ) | 538730 |
| 3 | TS=(("artificial intelligence" or "machine learning" or "deep learning" or "natural language processing" or "Artificial intelligence" or "Boltzmann machine*" or "Long short-term memory" or "Gated recurrent unit" or "Rectified linear unit" or Autoencoder or Backpropagation or "Multilayer perceptron" or Convnet or "Support vector machine" or "Random forest" or Lasso or Kernel or "Elastic net*" or Bayesian or "Naive bayes" or "Genetic algorithm")) | 1227270 |
| 4 | TS=((algorithm* or computeri* or "computer-based" or "computer based" or "machine-based" or "machine based" or "Computer assisted" or "Computer-assisted" or "Computer aided" or "Computer-aided" or integrat* or technolog* or digital or electron*) Near/4 ("decision support" or "decision-support" or "decision aid" or "decision-aid") ) | 8401 |
| 5 | TS=("computer assisted diagnosis" or "computer assisted" or "computer aided" or "decision support system*" or "decision making") | 664886 |
| 6 | #4 OR #5 | 667830 |
| 7 | #1 OR #2 OR #3 | 1652227 |
| 8 | #6 AND #7 | 66222 |
| 9 | TS=("Health Personnel" or "Healthcare Professionals" or "Health Care Professionals" or doctor* or nurse* or physician* or therapist* or pharmacist* or clinician* or practitioner* or Anaesthetist* or Audiologist* or physiologist* or cardiologist* or immunologist* or microbiologist* or dentist* or dietitian* or paramedic* or Gynaecologist* or midwives or midwife or obstetrician* or Ophthalmologist* or Optometrist* or Orthoptist* or Orthotist* or Paediatrician* or Pathologist* or Phlebotomist* or Physiotherapist* or Podiatrist* or chiropodist* or Prosthetist* or Psychiatrist* or Psychologist* or Psychotherapist* or Radiographer* or Radiologist* or surgeon* or "home care" or "care home*" or "nursing home*" or "home* for the aged" or "community health services" or "nursing care" or "point of care" or hospital*) | 3108589 |
| 10 | TS=(experience* or view or views or opinion* or accept* or attitude*) | 4910187 |
| 11 | #10 AND #9 AND #8 | 2267 |
